# HIV response financing challenges in Sub-Saharan Africa: barriers to achieving the 95-95-95 UNAIDS targets

**DOI:** 10.3389/fpubh.2025.1658229

**Published:** 2025-09-01

**Authors:** Benard W. Kulohoma, Colette A. Wesonga

**Affiliations:** Ortholog, Nairobi, Kenya

**Keywords:** 95-95-95 HIV/AIDS goals, response financing, Sub-Saharan Africa (SSA), challenges, HIV

## Abstract

Sub-Saharan Africa (SSA) faces a critical HIV financing crisis that threatens the 2030 goal of ending HIV as a public health threat. Despite accounting for 77% of weekly global new HIV infections, occurring among adolescent girls and young women, only 1% of annual global health spending is available for SSA, where 16% of the world’s population resides. This disparity undermines progress toward the UNAIDS 95-95-95targets for HIV diagnosis, treatment access, and viral suppression. Multiple converging health financing gaps exacerbate this crisis: Domestic resource mobilization remains inadequate, with only three African countries meeting the Abuja Declaration’s 15% health budget allocation target. Official development assistance (ODA) has declined by 70%, while reduced PEPFAR funding threatens treatment access for over 222,000 requiring daily treatment across seven high-burden countries. Health insurance coverage remains minimal, forcing up to 70% out-of-pocket health spending in some countries. Additionally, donor-driven vertical programs have fragmented health systems, while Africa produces only 3% of global pharmaceuticals despite bearing 23% of disease burden. These financing challenges extend beyond the HIV response, potentially causing 10.6 million additional tuberculosis cases and 2.2 million deaths during 2025–2030. Addressing this crisis requires coordinated action including strengthened domestic resource mobilization, innovative financing mechanisms, regional manufacturing capacity, and integrated health system governance. Urgent intervention is necessary to preserve decades of HIV prevention and treatment progress, particularly affecting the most vulnerable populations.

## Introduction

Sub-Saharan Africa (SSA) faces a critical HIV financing crisis that threatens to undermine global efforts to end HIV as a public health threat by 2030. Despite bearing 23% of the global disease burden, Africa receives only 1% of global health spending, where 16% of the world’s population resides (1). This disparity has profound consequences: life expectancy is 15.3 years shorter in Africa compared to Europe and 9.4 years below the global average (2), with fewer than half the number of doctors per 1,000 people compared to South-East Asia (3, 4).

The HIV epidemic continues to disproportionately affect SSA, with adolescent girls and young women (15–24 years) accounting for 77% of global new HIV infections recorded weekly (5–7). This group is affected by an interplay of poverty, violence, and lack of access to education that exacerbate vulnerability. Addressing these inequities is crucial to ending the HIV epidemic (8, 9).

The 95-95-95 HIV strategy targets 95% diagnosis, 95% treatment access among diagnosed, and 95% viral suppression among treated, shifting focus to person-centered care and expanding treatment as an essential public health intervention (10). However, the global community remains behind the joint United Nations programme on HIV/AIDS (UNAIDS) annual incidence target of less than 370,000 new infections in 2025 (11). Efforts to end the HIV epidemic as a public health threat by 2030 using a community-led response center on the most affected groups, aimed at eliminating gender inequity, legal and policy barriers to scale up prevention interventions (6).

### The health financing gap

The average health expenditure per capita by SSA country governments was just 1.5% of that spent in high-income countries (US $85 versus $5,767), and well below the global average of US $1,235 (12). This deficit has been further widened by the COVID-19 pandemic and leadership gaps, leading to inadequate staffing and medicines for a robust HIV response.

Achieving sustainable development goal (SDG) 3 requires an annual global budget of up to $240 billion (13–17). Current health threats require coordinated international responses that address the widening financing gap (17, 18).

## Challenges to sustainable HIV response financing

### Domestic resource mobilization challenges

While domestic resources for health in low- and middle-income countries (LMICs) increased by 50% between 2010 and 2019 and now exceed donor contributions, accounting for the majority of global HIV response funding (57% or US $10.6 billion) (19), this increase remains insufficient. The expected rate of domestic resource growth lags behind the gap left by the continuous decrease in donor assistance for health financing (20).

African countries committed to allocate at least 15% of national budgets to the health sector through the 2001 Abuja Declaration. However, only three countries (Rwanda, Botswana, and Cape Verde) have consistently met or exceeded this target, with most allocations remaining below 10% and some as low as 5% (21). Moreover, compounding external debt servicing deadlines of US $ 89 billion in 2025 leave little room to make health investments (21–24).

### The decline of official development assistance

Progress toward achieving universal health coverage (UHC) in Africa faces significant headwinds due to reductions in official development assistance (ODA), which represents 30% of total health expenditures (25). A 70% decline in ODA threatens to significantly reverse Africa’s positive health trends, including: the 50% reduction in child mortality under 5 years over the last three decades, expansion of immunization programs leading to near eradication of diseases like polio, and funding for the HIV response supporting over 18 million patients (10, 21, 26).

### The PEPFAR funding crisis

The termination of a significant component of the U.S. President’s Emergency Plan for AIDS Relief (PEPFAR) funding has severely dampened the HIV response, obscuring access to treatment for people living with HIV (27). PEPFAR is estimated to have saved 26 million lives and prevented 7.8 million infants from being born with HIV, halving HIV incidence since 2010 (27).

The decline in PEPFAR funding support has adversely affected HIV services at health facilities where over 222,000 patients received antiretroviral drugs daily, and up to 74,000 HIV deaths were predicted to occur in seven high-burden countries (Ethiopia, Kenya, Malawi, South Africa, Tanzania, Zambia, and Zimbabwe) during the executive stop work order period (28–31).

Children in SSA will be disproportionately impacted by HIV response funding disruptions, which could result in an almost three-fold increase in HIV incidence among children (32–34).

### Health insurance and out-of-pocket payment burden

Health insurance uptake remains very low in SSA, with existing national health insurance schemes in nascent phases, undermining the potential for treatment cost subsidization across countries (35, 36). Government-run health insurance schemes predominate in SSA, where large informal sectors with substantially low contributory capacity limit voluntary enrolment and contributions (37). Partnership between governments and the private sector are emerging and provide an opportunity to bridge the domestic resource mobilization gap (38).

Out-of-pocket health spending constitutes up to 70% of health expenditure in some countries, placing a significant burden on the most financially vulnerable populations (39, 40). Although the proportion of the population impoverished by out-of-pocket health spending at the extreme poverty line has decreased from 302 million (45.3% of total population) to 152 million (13.8%) globally over the last two decades, this progress has been slower than in other regions. Of concern is an increase in this proportion from 22.1% in 2000 to 44.2% in 2019 in SSA (40).

### Fragmented vertical programs

Donor-driven vertical programs have created fragmented health systems that strain local capacity. Conditional aid and short funding cycles undermine long-term planning, leaving African health systems vulnerable to abrupt shifts in donor priorities (19, 41). These vertical programs have fragmented health systems through duplicated functions and brain drain of health workers to better-funded donor programs, while weak regulatory environments compromise public-private partnerships (19, 42).

### Manufacturing and supply chain vulnerabilities

Africa produces only 3% of global drugs despite bearing 23% of the disease burden, with just 375 manufacturers compared to China’s 10,000 and India’s 5,000 (43). The continent uses 25% of global vaccines but produces only 1%, with COVID-19 exposing supply vulnerabilities and highlighting the need to expand local manufacturing beyond the current 11 vaccine manufacturers, where only South Africa and Senegal have full WHO pre-qualified production capabilities (44).

This manufacturing deficit severely impacts access to innovative HIV treatment and prevention tools. For instance, the availability of long-acting injectable HIV treatment remains limited in Africa. The biopharmaceutical company that has developed the twice-a-year injectable treatment, has pursued multiple access strategies, including tiered pricing and royalty-free licensing agreements with six manufacturers to produce low-cost generic versions for 120 low- and lower-middle-income countries (45–47), but local production capacity remains insufficient.

### Governance and leadership challenges

Poor leadership in Sub-Saharan Africa, characterized by corruption and weak governance, undermines health systems financing through ineffective policies, increased costs, and ultimately poor population health outcomes (39). The complexity of the healthcare ecosystem requires ministries of health to strengthen governance to integrate diverse actors and build capacity for contracting civil society organizations for community-level interventions like HIV/AIDS awareness (4).

### Consequences for the 95-95-95 targets

These financing challenges undermine progress toward attaining the UNAIDS 95-95-95 targets. Reduced funding for testing and diagnosis programs, and community outreach limits the ability to identify undiagnosed individuals, particularly in hard-to-reach populations (48). ART supply disruptions and facility closures due to funding cuts prevent diagnosed individuals from accessing life-saving treatment (49). Inadequate funding for monitoring systems, adherence support, and management of opportunistic infections compromises viral suppression rates among those on treatment (50–52).

### Broader health security implications

The financing crisis extends beyond HIV to threaten broader health security. In 26 high-burden countries accounting for 80% of the global TB burden, reduced ODA and PEPFAR funding support for TB response is estimated to lead to 10.6 million additional TB cases and 2.2 million additional TB deaths during 2025–2030, reversing previous gains (53, 54). This ultimately poses a global health threat through disease transmission (30).

The reduction in ODA funding coincides with a 41% increase in infectious disease outbreaks, including Mpox, cholera, measles, and Ebola, alongside climate change and humanitarian emergencies causing food insecurity and threatening livelihoods (21, 55).

## The path forward: financing solutions and recommendations

### Strengthening domestic resource mobilization

Health financing is essential to enable resilient healthcare systems capable of accomplishing the SDGs, including universal health coverage by 2030 (39). Countries that achieve appropriate health financing will eradicate preventable morbidity and mortality, adequately respond to public health emergencies, reduce disability-adjusted life years (DALYs), and stir long-term economic growth (56). Closing the health financing deficit requires new, robust investments with acceptable risk-adjusted returns (16). An optimal integrated approach will combine fiscal programming, planning, human resources, and capacity building.

At present, government-run health insurance schemes predominate in Sub-Saharan Africa, where the majority are employed in the informal sector with low contributory capacity impeding voluntary enrolment and contributions, and making it difficult to achieve universal health coverage. Partnership between governments and the private sector are emerging and provide an opportunity to bridge the domestic resource mobilization gap (38). However, coordinated public–private partnerships (PPPs) and public institutional reforms are necessary to improve health-sector outcomes. A risk-adjusted return approach that takes into account the potential profit by private sector investors, while also considering the degree of acceptable public risk versus the long term benefits due to economic volatility should be considered. This implies that PPPs are only implemented when they represent the most cost-effective solutions compared to other available options (57). An example is the PPP in Lesotho, which bears a disproportionate HIV disease burden, to replace a run-down 100 year-old hospital with a new one with care clinics that improved access, care services and expanded population coverage of HIV prevention (58, 59).

Success requires comprehensive regulatory frameworks that clearly define roles and responsibilities of the public and private sector actors to ensure accountability, transparency and ease of dispute resolution enabling high quality standard deliverables. This entails development of standardized contracts to reduce negotiation time and protect public interests, thereby avoiding corruption loopholes during procurement. Centralized units to coordinate the PPPs will streamline planning, implementation and oversight, and ensure integration of health policies and alignment with ministries of finance during the fiscal planning cycles (60). Governments will ensure they can afford costs from beginning to end without compromising other public social obligations or reducing investments in other progressive healthcare areas that are not part of the PPPs. In the end this will enable implementation of performance-based contracts linking payments to deliverable quality and financial sustainability (59).

### Innovative financing mechanisms

Effective health financing mechanisms are essential to provide quality and affordable health services to all (61). These services include prevention of new infections, treatment provision, and support that helps with illness management to improve quality of life. For those living with HIV, key services include testing, prevention (including PrEP and condom use), treatment (ART), and support services like counseling and management of opportunistic infections (39).

A common practice has been to use development bank-backed guarantees Africa Development Bank (AFDB), World Bank, and International Finance Corporation (IFC) or insurance mechanisms to mitigate political, currency, or demand risks, and attract and maintain private sector investment, including healthcare financing. Countries like Rwanda, Botswana and Cape Verde have structured their health financing to maximize resources, ensure sustainability, and to align their own priorities, as opposed to donor-driven agendas. In Rwanda, a community-based health insurance (Mutuelles de Santé) scheme pools income-adjusted premiums at the community level, with the government subsidizing premiums for the poorest (62, 63). Moreover, health facilities receive funding based on performance indicator incentivizing quality and efficiency in service delivery (64). This enables universal health coverage and financial protection, while reducing individual costs. Botswana uses diamond revenue and sin taxes (on tobacco and alcohol) to fund health (65). Cape Verde earmarks a proportion of value-added tax (VAT) and social security contributions for health financing (66, 67).

Beyond public-private partnerships in infrastructure, innovative insurance partnerships are emerging across SSA to address coverage gaps. Private sector insurance providers are increasingly partnering with global insurers based in economically developed countries to expand reach and improve uptake across SSA. These partnerships leverage international expertise in risk management and digital platforms while adapting products to local contexts and affordability constraints, offering a pathway to complement government schemes and reach underserved populations in the informal sector.

### Regional manufacturing and supply chain strengthening

Although the healthcare and wellness business sector in Africa is estimated to be worth $259 billion by 2030, with a potential to create 16 million jobs (68), progress is still slow. Deliberate choice via transformative policy implementation by governments is necessary to bridge this gap. New African vaccine manufacturers face competition from established low-cost suppliers, requiring multi-stakeholder support including sustainable investment, clear demand signals from SSA countries, enhanced regulatory capacity, and coordinated international assistance across research, manufacturing, and regulatory frameworks. In South Africa, the Biovac institute provides an example of a PPP to localize and strengthen vaccine and biologicals manufacturing to enable self-reliance and health security (69). The South African government used a PPP model to co-invest in vaccine production and distribution, reducing stockouts and stabilizing pricing. Long-term benefits of this investment overshadow the required initial investment. Fiscal planning changes to reduce tax on raw materials for the health sector, diagnostic services, and work permits fees for health experts required for technology transfer will significantly strengthen regional health capacity.

Rwanda’s government is also developing a pharmaceuticals manufacturing ecosystem in partnership with the private sector and has also taken into consideration the need to support the development of its regulatory agency (70).

There is an urgent need to finance sustainable vaccine and pharmaceutical manufacturing in SSA, emphasizing investment, collaboration, and capacity building to enhance regional supply resilience and meet future health challenges (71). Gavi’s African Vaccine Manufacturing Accelerator (AVMA) provides a turning point to incentivize investors to support Africa-led solutions to expanding access to vaccines and other therapeutics, such as antibodies (72).

### Health system integration and governance

Development partners should align disease-specific programs with national systems rather than creating parallel structures. African governments should leverage donor support to strengthen institutional capacity aligned with public interests to ensure sustainability (38). Ministries of health must strengthen governance to integrate diverse actors and build capacity for contracting civil society organizations for community-level interventions. Countries must align external assistance with national health priorities, budgetary frameworks, and accountability mechanisms to foster coherent partnerships and prevent system fragmentation.

Health financing solutions for universal health coverage in SSA require tailored, country-specific approaches rather than generalized models (42).

## Conclusion

New equitable and resilient strategies in SSA are required to lead the response toward attaining the 95-95-95 UNAIDS targets and ensure health sovereignty (6, 73, 74). The current financing crisis, exacerbated by declining donor support and inadequate domestic resource mobilization, threatens to undermine decades of progress in HIV prevention and treatment.

Global HIV funding has remained stagnant since 2017, with increasing pressure on SSA countries to domestically finance their responses. Recent international aid reductions threaten to undermine progress unless PEPFAR support is reinstated and countries can absorb 30–90% of other funding cuts through improved efficiencies and domestic investment (75). Even with PEPFAR reinstatement, many countries will struggle to adequately resource their HIV responses due to competing health and development priorities, potentially jeopardizing gains in controlling the epidemic.

Addressing these challenges requires urgent, coordinated action across multiple stakeholders to develop sustainable financing mechanisms, strengthen health systems, and ensure that the promise of ending HIV as a public health threat by 2030 remains achievable for the most affected populations in Sub-Saharan Africa.
